# Fulminant hepatitis following COVID‐19 vaccination: A case report

**DOI:** 10.1002/ccr3.6066

**Published:** 2022-07-19

**Authors:** Mohammad Barary, Athena Sharifi‐Razavi, Nasser Rakhshani, Terence T. Sio, Soheil Ebrahimpour, Mana Baziboroun

**Affiliations:** ^1^ Student Research Committee, Virtual School of Medical Education and Management Shahid Beheshti University of Medical Sciences Tehran Iran; ^2^ Students' Scientific Research Center (SSRC) Tehran University of Medical Sciences Tehran Iran; ^3^ Clinical Research Development Unit of Bou‐Ali Sina Hospital Mazandaran University of Medical Sciences Sari Iran; ^4^ Department of Pathology, Gastrointestinal and Liver Diseases Research Centre, Firoozgar Hospital Iran University of Medical Sciences Tehran Iran; ^5^ Department of Radiation Oncology Mayo Clinic Phoenix Arizona USA; ^6^ Infectious Diseases and Tropical Medicine Research Center, Health Research Institute Babol University of Medical Sciences Babol Iran

**Keywords:** AstraZeneca, COVID‐19, hepatitis, SARS‐CoV‐2, vaccine, fulminant

## Abstract

The common side effects of COVID‐19 vaccination were mostly self‐restricted local reactions that quickly resolved. Nevertheless, rare autoimmune hepatitis cases have been reported in some vaccinated with mRNA COVID‐19 vaccines. This article presents a young man who developed fulminant hepatitis a few days after vaccination with the first dose of the AstraZeneca COVID‐19 vaccine. A 35‐year‐old man was admitted to our hospital with generalized weakness, abdominal pain, and jaundice. He received the first dose of the AstraZeneca COVID‐19 vaccine 8 days earlier. He was admitted to the hospital with a chief complaint of abdominal pain. On admission and because of his high D‐dimers, low platelet count, and low Fibrinogen level, vaccine‐induced immune thrombosis thrombocytopenia was suspected, which was ruled out later. Then, after a surge in his liver function tests, decreasing platelet, and abnormal clotting tests, fulminant hepatitis was considered for this patient. Several bacterial, viral, and autoimmune etiologies were then suspected, with all ruled out. Thus, fulminant hepatitis secondary to his AstraZeneca COVID‐19 vaccine was confirmed. Unfortunately, he died 3 days later of disseminated intravascular coagulopathy, after which a liver necropsy was performed, indicating drug/toxin‐induced hepatitis.

## Abstract 2

Although rare, fulminant hepatitis could be a fatal adverse event following COVID‐19 vaccination, mimicking signs and symptoms and the laboratory workup of vaccine‐induced immune thrombosis thrombocytopenia. If suspected, liver function tests and coagulation assays should be performed, and high‐dose corticosteroids must be started immediately.

## 1 INTRODUCTION

The common side effects of COVID‐19 vaccination were mostly self‐restricted local reactions that quickly resolved.^1^ Nevertheless, rare autoimmune hepatitis cases have been reported in some vaccinated with mRNA COVID‐19 vaccines.^2^, ^3^ This article presents a young man who developed fulminant hepatitis a few days after vaccination with the first dose of the AstraZeneca COVID‐19 vaccine.

## 2 CASE PRESENTATION

**FIGURE 1:**
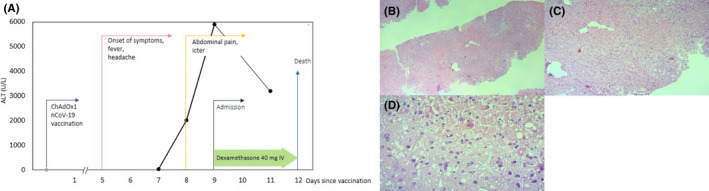
ALT trend and histological findings. (A) Trends of plasma ALT. (B) Low‐magnification (40×). (C) Medium‐magnification (100×). (D) High‐magnification (400×) of liver necropsy demonstrating multifocal confluent necrosis in liver lobule, mild lymphocytic infiltration in sinusoids, and between hepatocytes

A 35‐year‐old man was admitted to our hospital with generalized weakness, abdominal pain, and jaundice. He received the first dose of the AstraZeneca COVID‐19 vaccine 8 days earlier. Five days after vaccination, the patient experienced fever and headache. Despite the improvement of fever, his abdominal pain was exacerbated, and loss of appetite, jaundice, and vomiting was also manifested. Thus, he was admitted to our center. He had a history of psychological problems under control with paroxetine and sodium valproate. His laboratory results on admission are summarized in Table 1. According to the high D‐dimers, low platelet count, and low fibrinogen level, vaccine‐induced immune thrombosis thrombocytopenia was suspected. Therefore, high‐dose dexamethasone 40 daily, IVIG 1 mg/kg for 2 days, and rivaroxaban 15 mg daily were started for the patient. Abdominal ultrasonography showed a grade I fatty liver disease, normal gall bladder, and pancreas with mild effusion in subdiaphragmatic space. Also, no intra‐ or extra‐hepatic biliary dilatation was observed. Color Doppler ultrasonography revealed normal portal and hepatic vein and inferior vena cava. The laboratory results of his second hospitalization day were indicative of a surge in his hepatic enzymes, decreasing platelet, prolonged partial thromboplastin time, and increased international normalized ratio (Table 1) (Figure 1A).

**TABLE 1:** Clinical and laboratory characteristics of the patient on admission, and during the hospitalization

Characteristic	Reference value	Findings	
		On admission	During hospitalization
Age (year)		35	
Sex		Male	
Preexisting conditions		Psychological problems	
Time from vaccination to admission (day)		8	
Symptoms and signs		Severe abdominal pain, loss of appetite, jaundice, icteric sclera, and vomiting	
Platelet count (per μl)	150,000–400,000	50,000	27,000
d‐dimer (ng/ml)	<500	15,000	>18,000
Fibrinogen (mg/dl)	200–400	179	153
INR		1	1.5
PTT (s)	25–45	40	51
LDH (U/L)	<480	4800	>5400
CRP (mg/L)	<10	66	86
ESR (mm/h)	<20	3	5
Bilirubin total (mg/dl)	<1.2	4.7	15.3
Bilirubin direct (mg/dl)	<0.4	1.5	3.7
AST (U/L)	5–40	1000	4700
ALT (U/L)	10–55	2000	5900
ALP (U/L)	24–147	461	713

Abbreviations: ALP, Alkaline phosphatase; ALT, Alanine aminotransferase; AST, Aspartate aminotransferase; CRP, C‐reactive protein; ESR, Erythrocyte sedimentation rate; INR, International normalized ratio; LDH, Lactate dehydrogenase; PTT, Partial thromboplastin time.

Furthermore, laboratory results were negative for hepatitis A, B, C, and E viruses, HIV, EBV, HSV‐1, and HSV‐2. Anti–nuclear antibody, anti–smooth muscle antibody, and SARS‐CoV‐2 reverse transcriptase‐polymerase chain reaction were also negative. Hence, fulminant hepatitis was suspected in the patient. Due to a lack of clinical improvement, he became a liver transplant candidate. Unfortunately, after 3 days of hospitalization, he expired as of disseminated intravascular coagulation, after which a liver necropsy was performed, indicating drug/toxin‐induced hepatitis (Figure 1B–D).

## 3 DISCUSSION

Vaccines have been shown to trigger an immune response leading to a broad spectrum of autoimmune diseases.^4^ The spike glycoprotein of SARS‐CoV‐2 or adenoviral vector spike protein vaccines share genetic similarities with a large heptapeptide human protein, so this is an additional factor that can trigger autoimmune disease after vaccination due to molecular mimicry.^5^ In our patient viral markers were negative, and there was no history of drug or toxin exposure. Unfortunately, our patient died quickly after hospitalization, making our laboratory tests incomplete in determining the etiology of his fulminant hepatitis. On the other hand, the patient was being treated with paroxetine and sodium valproate for his psychological illnesses that might have increased the chance of fulminant hepatitis following vaccination. Since these side effects are infrequent, such cases should not discourage people from being vaccinated against COVID‐19, yet physicians must be vigilant for such potential adverse events.

## AUTHOR CONTRIBUTION

The case was diagnosed and followed up by NR and MB. SE and MB conceived and planned the case report, MB, ASR, and TTS wrote the manuscript, and MB and TTS edited the first draft and provided substantial revision. The final version was read, corrected, and approved by all authors. All co‐authors take full responsibility for the integrity of the case study and literature review.

## CONFLICT OF INTEREST

TTS reports that he provides strategic and scientific recommendations as a member of the Advisory Board and speaker for Novocure, Inc. and also as a member of the Advisory Board to Galera Therapeutics, which are not in any way associated with the content or disease site as presented in this manuscript. All other authors have no relevant financial interests to be declared.

## CONSENT

Written informed consent was obtained from the patient for publication of the current case report.

## Data Availability

The data that support the findings of this study are available from the corresponding author upon reasonable request.
